# Development of an algorithm for determining smoking status and behaviour over the life course from UK electronic primary care records

**DOI:** 10.1186/s12911-016-0400-6

**Published:** 2017-01-05

**Authors:** Mark D. Atkinson, Jonathan I. Kennedy, Ann John, Keir E. Lewis, Ronan A. Lyons, Sinead T. Brophy, on behalf of the DEMISTIFY Research Group, Ashley Akbari, Mark D. Atkinson, Sinead T. Brophy, Joanne C. Demmler, Martin L. Heaven, Jonathan I. Kennedy, Arron S. Lacey, Daniel S. Thayer, Samantha L. Turner, Angharad Walters

**Affiliations:** 1 Farr Institute, Swansea University Medical School, Swansea, SA2 8PP UK; 2 Prince Philip Hospital, Hywel Dda Health Board, Llanelli, UK

**Keywords:** Smoking status, Smoking cessation, Data linkage, SAIL databank

## Abstract

**Background:**

Patients’ smoking status is routinely collected by General Practitioners (GP) in UK primary health care. There is an abundance of Read codes pertaining to smoking, including those relating to smoking cessation therapy, prescription, and administration codes, in addition to the more regularly employed smoking status codes. Large databases of primary care data are increasingly used for epidemiological analysis; smoking status is an important covariate in many such analyses. However, the variable definition is rarely documented in the literature.

**Methods:**

The Secure Anonymised Information Linkage (SAIL) databank is a repository for a national collection of person-based anonymised health and socio-economic administrative data in Wales, UK. An exploration of GP smoking status data from the SAIL databank was carried out to explore the range of codes available and how they could be used in the identification of different categories of smokers, ex-smokers and never smokers. An algorithm was developed which addresses inconsistencies and changes in smoking status recording across the life course and compared with recorded smoking status as recorded in the Welsh Health Survey (WHS), 2013 and 2014 at individual level. However, the WHS could not be regarded as a “gold standard” for validation.

**Results:**

There were 6836 individuals in the linked dataset. Missing data were more common in GP records (6%) than in WHS (1.1%). Our algorithm assigns ex-smoker status to 34% of never-smokers, and detects 30% more smokers than are declared in the WHS data. When distinguishing between current smokers and non-smokers, the similarity between the WHS and GP data using the nearest date of comparison was κ = 0.78. When temporal conflicts had been accounted for, the similarity was κ = 0.64, showing the importance of addressing conflicts.

**Conclusions:**

We present an algorithm for the identification of a patient’s smoking status using GP self-reported data. We have included sufficient details to allow others to replicate this work, thus increasing the standards of documentation within this research area and assessment of smoking status in routine data.

**Electronic supplementary material:**

The online version of this article (doi:10.1186/s12911-016-0400-6) contains supplementary material, which is available to authorized users.

## Background

Smoking is one of the major causes of morbidity and mortality in the United Kingdom and was responsible for 19% of total deaths in 2005 and 12% of disability adjusted life years lost in 2002 [1]. A patient’s smoking status can be a crucial factor in epidemiological studies as a confounder/covariate or as the primary exposure variable. Recent changes in access to routinely collected health care data has resulted in greater utilisation of primary care records as a source for smoking status in a range of studies [2–8].

The recording of smoking status, which should take place during registration of all new patients is variable [9] and a systematic review of the scope and quality of primary care data indicated that diagnostic and lifestyle data are populated less than prescription data [10]. Overcoming this issue is necessary to acquire accurate information on a patient’s smoking status. The implementation of the UK’s Quality Outcomes Framework (QOF) in 2004 offered financial incentives to GPs for improving the recording of specific outcomes, including smoking status [11–13]. During this period, guidelines were implemented advising on how to record smoking status in relation to age, certain medical conditions, such as chronic obstructive pulmonary disease and asthma, and previous records [14], consequently moving towards greater standardisation of records. In spite of these efforts, individual GPs and practice nurses, working within guidelines set at many levels (national, health board and practice) are recording smoking status at various encounters with patients.

While smoking status codes are often used in isolation within epidemiological studies, additional information is available. This information can be applied to triangulate and improve the assignment of smoking status. The majority of smokers attempt to quit without pharmacotherapy, exemplified by a study of the use of prescription information for smoking cessation medications within a GP database [15]. Prescriptions from The Health Improvement Network (THIN) GP database were validated against a database of pharmacists’ dispensing returns, showing generally high comparability for Nicotine Replacement Therapy (NRT) prescriptions for 2004 and 2005. Dispensed prescriptions exceeded issued prescriptions by about 8%. The authors attributed this to prescriptions issued in the community and not therefore recorded in the GP database. The addition of smoking cessation therapy prescription data is a valuable augmentation to smoking status codes. Furthermore, there are numerous codes in GP databases indicating such concepts as; the wish to quit smoking, referrals to cessation therapy, generation of reminder letters, and placement of patients on cessation education schemes etc. These codes have been employed in at least one study [16]. The appropriate application of these codes assists in the identification of a patient’s correct smoking status.

In bespoke and relatively small scale pharmacotherapy and biological studies, smoking status, usually strictly defined with self-reporting, is often validated with chemical testing, such as exhaled carbon monoxide, salivary or urinary cotinine over sustained periods. The Russell standard [17] is the most widely accepted test. Subsequently, it has been proposed as a measure of success of community and hospital-based Stop Smoking Services, however, biological validation is not feasible in the general population attending primary care. The epidemiological literature is frequently deficient in detail concerning how smoking status is determined. Frequently, authors apply codes to identify smoking status at a particular date, but do not employ data across the life course, thus enabling the determination of ambiguities as opposed to genuine changes in behaviour (such as relapse/recurrent quit attempts). The authors of one study [16] have made this adjustment. Generally, however, the literature rarely lists the detailed codes and algorithms used resulting in a lack of transparency, multiple reinventions of the wheel and an inability to replicate findings.

The objectives of this paper are fourfold;

1. To combine codes on smoking status, smoking intensity, prescriptions for cessation therapies and administrative codes for cessation regimes to acquire information about smoking status.
2. To take a life course approach to changes in smoking status and smoking behaviour in GP records.
3. To develop an improved algorithm to define smoking status, incorporating these various codes.
4. To compare the results of the algorithm by linking individual GP data to another source where smoking status is self-reported for the same individuals.

## Methods

This research used de-identified linked patient information from the SAIL databank. We developed an algorithm using data from the GP database. Responses from the participants of the Welsh Health Survey were linked with their corresponding SAIL records. We used the algorithm to determine a patient’s smoking status at the time of the WHS questionnaire response. Thus we could directly compare, at patient level, smoking status derived from the GP data with that recorded in their response to the WHS questionnaire.

### Data Sources

#### The SAIL databank

The SAIL databank was established to bring together, link and anonymise the widest possible range of person-based data to support health research, funded by Health and Care Research Wales (Welsh Government). The system was established after wide consultation with a number of stakeholders including; several departments of the Welsh Government, the British Medical Association (BMA), The Royal College of General Practitioners, the National Research Ethics Service (NRES) and other professional bodies [18]. Effective privacy protection is achieved by using a split-file approach with multi-party encryption and restriction on linkage to third party datasets in uncontrolled environments [19]. The SAIL databank operates within a robust series of guidelines in line with the Caldicott principles and the National Information Governance Board for Health and Social Care [18]. Data submitted by providers are de-identified by using a split file approach. This is done by submitting two files with a linking field to two destinations. A demographic file (with name, address, NHS number) is sent to a trusted third party (The NHS Wales Informatics Service NWIS). The other file, with clinical information is sent to the SAIL Databank. NWIS replaces the demographic information with an anonymised linking field after matching to a population file and sends this file to the SAIL Databank. The two are then merged.

The SAIL databank is housed at Swansea University. The research team is based in the Centre for Improvement in Population Health through E-records Research (CIPHER), one of the four centres comprising the Farr Institute of Health Informatics Research.

The Welsh Demographic Service (WDS) file contains all people registered with a GP in Wales. This provides a backbone for linkage of datasets within the SAIL databank. The GP dataset, which at the time of analysis included approximately 50% of GP practices across Wales with data on over two million people. This contains clinical and administrative data collected during consultations as well as all prescriptions issued by the GP. Individual GP practices give consent to have their data submitted to the SAIL databank.

The data held in the SAIL system are anonymised and have been obtained with the permission of the relevant Caldicott Guardian and Data Protection Officer of the data providers. The National Research Ethics Service has stated that no ethical review is required. Ethical review is generally not required when anonymised data alone are used [20]. We gained approval from the Information Governance Review Panel, an independent body comprising representatives from the British Medical Association (BMA), National Research Ethics Service (NRES), Public Health Wales, the NHS Wales Informatics Service (NWIS) and the SAIL Consumer Panel. This body reviews all proposals to use SAIL data to ensure that they are appropriate and in the public interest [21].

#### Welsh Health Survey

The 2013 and 2014 Welsh Health Surveys [22, 23] were undertaken by a home visit through which questionnaires were completed by the adults in the household.

The questions below enquire about the participant’s smoking status.

1. I smoke daily.
2. I smoke occasionally but not every day.
3. I used to smoke daily but do not smoke at all now.
4. I used to smoke occasionally but do not smoke at all now.
5. I have never smoked.

From the responses to this question, the following smoking status variable is derived; one or two – smoker, three or four – ex-smoker, five – never smoker.

The participants were asked to consent was sought for linkage to the SAIL databank. Linkage was carried out by a trusted third party, the NHS Wales Informatics Service.

#### Recording of smoking status in the GP database

GPs and all other primary care personnel (district nurses, healthcare assistants, practice nurses) are encouraged to ask patients about smoking status at every appropriate opportunity, starting at the registration of a new patient. Guidelines and principles are summarized by Sharma [14]. If a patient is recorded as an ex-smoker in three consecutive years, they do not need to be asked again. Patients aged 26 and coded as never having smoked after 25 need no further recording. Patients between 15 and 25 recorded as never smokers should be reviewed every 15 months.

#### Read codes for smoking status

The GP database currently contains Version 2 5-byte Read codes. A detailed list of Read codes contributing to this algorithm are given in Additional file 1: Table S1, also a summary of the “NEVER_SMOKER”, “EX_SMOKER” or “SMOKER” classes are presented in Table 1, and the smoking intensity codes are shown in Table 2.

**Table 1:** Breakdown of the sub-groups within the smoking status groups

Group	Sub Group	Count of codes within group
NEVER_SMOKER	Never smoked tobacco	2
EX_SMOKER	Ex smoker (general)	7
	Ex smoker intensity prior to quitting	5
	Ex smoker type (non cigarette)	4
	Ex smoker (cigarette only)	1
	Ex smoker admin	1
SMOKER	Nicotine Replacement Therapy	91
	Passive cessation	16
	Smoker (general)	14
	Smokers unwilling to quit	12
	Active Cessation	12
	Administrative	8
	Advice	7
	Smoking Value	6
	Therapy	5
	Smoking intensity	5
	Smoker type (non cigarette)	5
	Nicotine dependence	2
	Toxicity	2
	Smoker type (cigarette only)	1

**Table 2:** Classification of smoking intensity of a smoker. Zero counts may represent non smoker or smoker,﻿ dependent on GP’s input

Class	Types of codes
Trivial Smoker	Trivial Smoker, Ex Trivial Smoker
Light Smoker	Light Smoker, Ex Light Smoker, Tobacco/Cigarette consumption less than 10 and greater than 0, Over 60 min before first cigarette on waking.
Medium Smoker	Medium Smoker, Ex Medium Smoker, Tobacco/Cigarette consumption less than 20 and greater than 0, Over 30 min before first cigarette on waking.
Heavy Smoker	Heavy smoker, Ex heavy smoker, Tobacco/Cigarette consumption less than 40 and greater than 0, Over 5 min before first cigarette on waking.
Very Heavy Smoker	Very Heavy Smoker, Ex Very Heavy Smoker, Tobacco/Cigarette consumption more than 40, under 5 min before first cigarette on waking.

There are two codes associated with “NEVER_SMOKER”, these are the never smoked tobacco code and a review code (non-smoker annual review). The “EX_SMOKER” category is split into multiple groups including; self-described former smokers of cigarettes or other forms of smoking tobacco (cigars, pipes, etc.), as well as former smoking intensity and administrative codes. Administrative codes indicate referral for cessation therapy, treatment by the GP or non-attendance.

The “SMOKER” Read codes incorporate several groups including; nicotine replacement therapy, codes associated with involvement/refusal of stop smoking services, smoking via non-cigarette forms of tobacco inhalation, nicotine dependence/toxicity, and smoking intensity.

However, there are complications with the coding of smoking status, including the use of a parent code which, on its own carries no information [24]. Another problem is the use of ‘never-smoker’ status after previous instances of codes implying smoking [25].

#### Algorithm development

This smoking algorithm has been developed to be able to correctly assign smoking status to a patient at a census date.

There are three starting categories; never smoker, ex smoker and smoker. The GP record for a patient is scanned and each smoking related Read code is assigned to one of these categories. For each patient, the first and last date of a code in each category is recorded. So we have FIRST_EX (the first recorded date of an ex smoker code), LAST_EX (the last recorded date of an ex smoker code), FIRST_NEVER and LAST_NEVER for the never smoker category and FIRST_SMOK and LAST_SMOK for the smoker category.

**Fig. 1:**
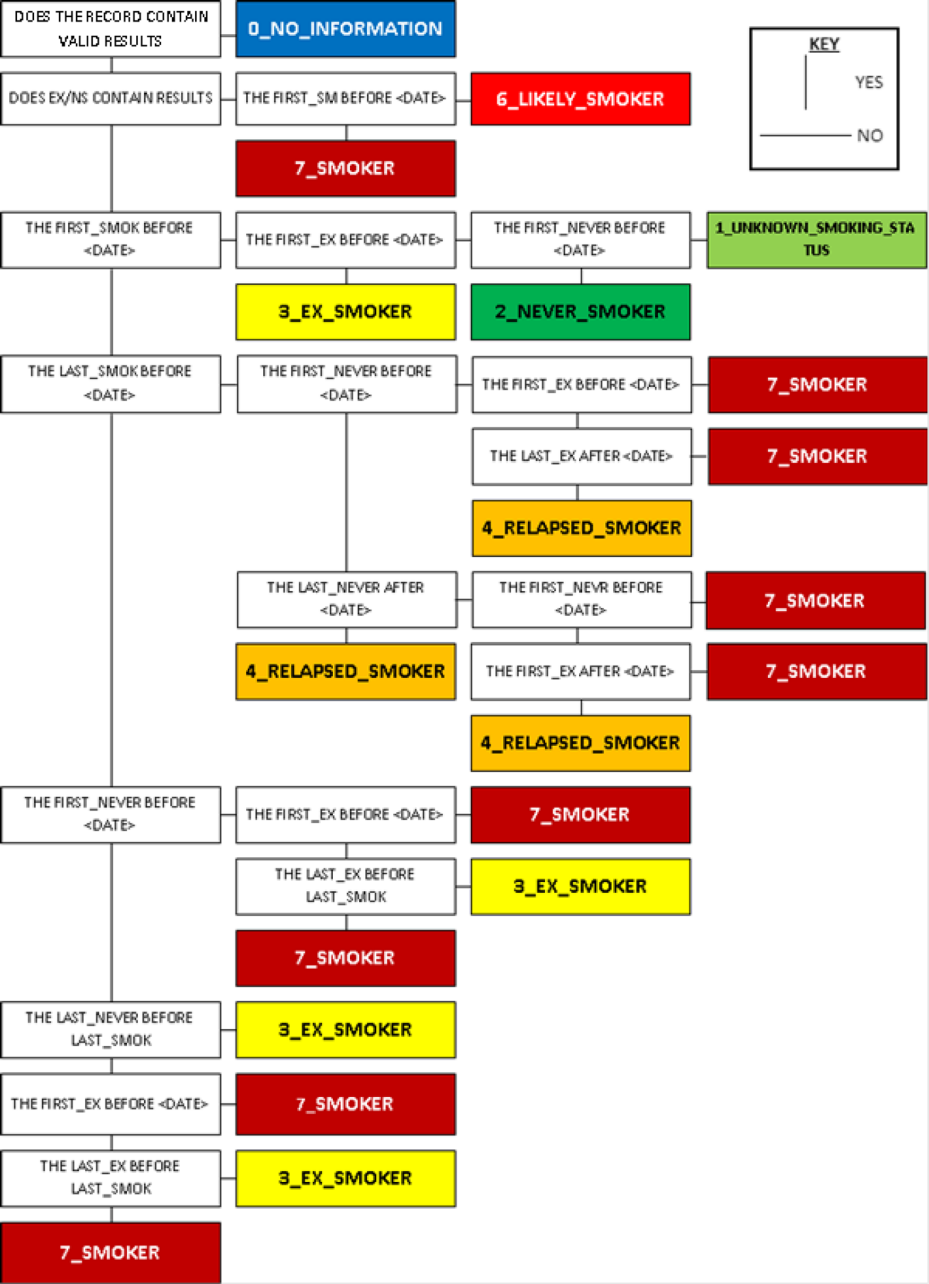
Full smoking algorithm flow diagram

The full Structured Query Language (SQL) flow diagram is shown in Fig. 1. Here, a number of different classes are considered:

- “NO_INFORMATION” ○ If a patient is identified as having “NO_INFORMATION” then there are no Read codes associated with smoking status for the patient’s entire GP record. This class allows greater understanding of the coverage of smoking status within the population of interest.
- “UNKNOWN_SMOKING_STATUS” ○ The patient has no smoking status Read codes prior to the event date, but has a smoking status after the event date. The purpose of this designation is to assist in determining if the event date chosen is suitable to give a good coverage of smoking status.
- “NEVER_SMOKER” ○ This patient has “NEVER_SMOKER” related codes prior to the event. In addition to no “EX_SMOKER” or “SMOKER” related Read codes, recorded prior to the event.
- “EX_SMOKER” ○ The patient is a self-identified former smoker and those that categorise themselves as never smokers but have a past-coded experience of smoking, are identified as ex-smokers.
- “RELAPSED_SMOKER” ○ This status is for events that have the “SMOKER” and “EX_SMOKER” and/or “NEVER_SMOKER” overlapping during the event date.
- “LIKELY_SMOKER” ○ The patient only has “SMOKER” related classes but none prior to the event date. This class was determined as necessary (owing to a result of a consequence of) the initiation of smoking during teenage years or earlier, however, recording of smoking status by a GP may not arise/transpire until years after initiation.
- “SMOKER” ○ The patient is a current smoker. This is identified by utilising Read codes associated with cessation, smoking as a smoking status or smoking intensity.

**Fig. 2:**
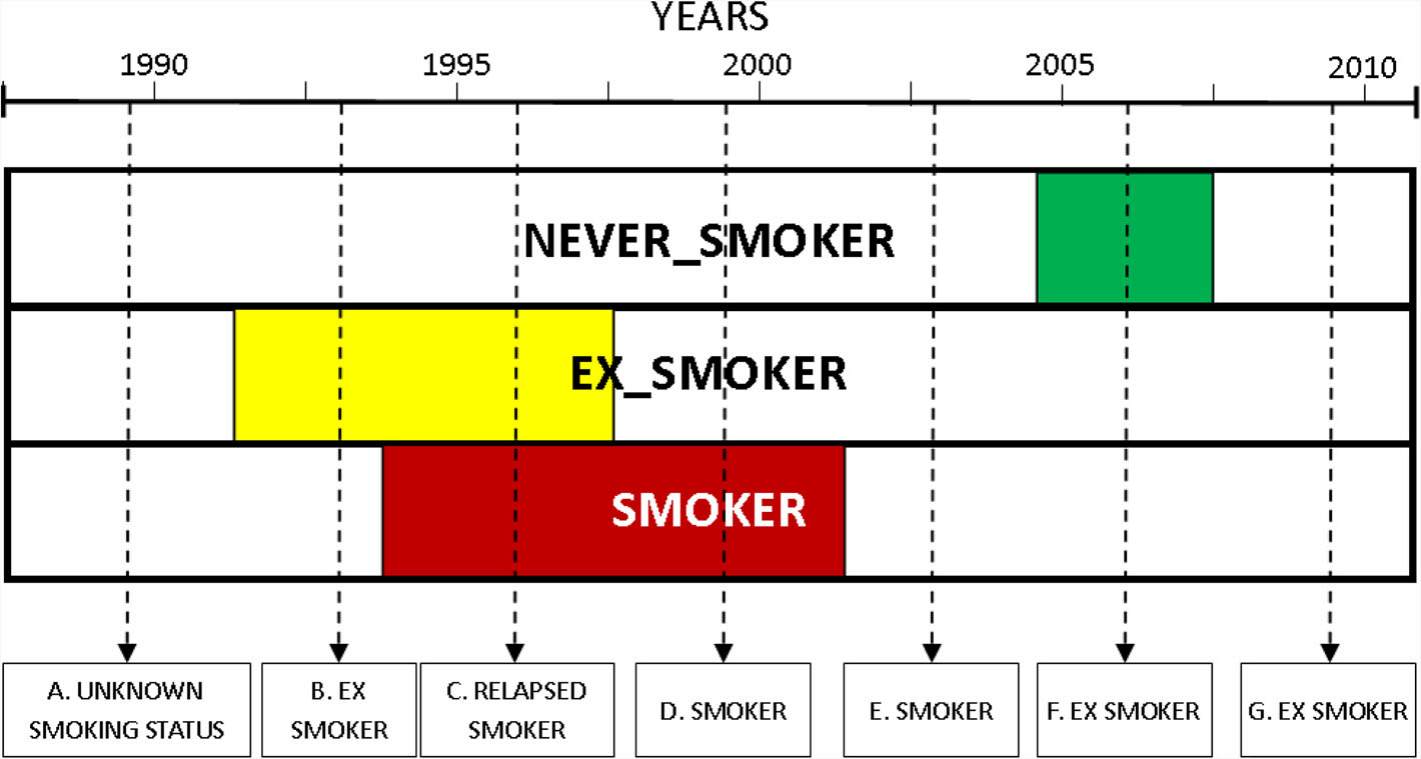
Derivation of smoking status categories. This represents a single person through time. The top row (NEVER_SMOKER) shows the first and last dates as the start and end of the green bar. The second row (EX_SMOKER) shows the first and last dates as the start and end of the yellow bar. The third row (SMOKER) shows the first and last dates as the start and end of the red bar

Figure 2 illustrates how a smoking status classification is given to an individual at various points in their history. Census date A is before the first recorded date of “NEVER_SMOKER”, “EX_SMOKER”, or “SMOKER” and therefore the classification will be “UNKNOWN_SMOKING_STATUS”.

Census date B is after the first recorded date for “EX_SMOKER”, therefore the designation is “EX_SMOKER”.

The third census date, C, in the history of this patient is characterised by the “EX_SMOKER” and “SMOKER” classifications having start dates before the event and end dates after the event. This overlapping of classes is identified as “RELAPSED_SMOKER” because they are likely to have relapsed from not smoking during this period.

At census date D the only category with a class before and after the event is “SMOKER”; therefore the status is “SMOKER”.

At census date E the patient is categorized as “SMOKER” because the time point does not overlap any other classes but the closest recorded date to this point is the last “SMOKER” date.

At census date F the “NEVER_SMOKER” first date is before the census date and the last recorded date is after the census date. In isolation this would lead to a status of “NEVER_SMOKER”, however, as this patient has a history of being an “EX_SMOKER” and/or a “SMOKER” prior to this event, thus the status assigned will be “EX_SMOKER”.

Census date G arises after all recorded class dates and since the most recent status is “NEVER_SMOKER” the class assigned would be “NEVER_SMOKER”. However, there is recorded history of “EX_SMOKER” and/or a “SMOKER” status causing the status to be “EX_SMOKER”.

### Comparison with another dataset

Using the SAIL privacy protecting data linkage system, a comparison was made between assessments of smoking status for the same individuals in the Welsh Health Survey (WHS) and GP data. Data provided by people who had given their consent for their data to be linked in the 2013 and 2014 Welsh Health Surveys was linked to the SAIL GP data. Where they had any GP data they were included in the analysis, although some of them did not have data on smoking status. There was no overlap between the two WHS annual surveys. The algorithm was used to estimate the smoking status at the date of the appropriate survey.

Results from the smoking algorithm were compared to questionnaire results taken from the WHS surveys.

Comparison of the WHS data and GP data was carried out using three algorithms.

1. The full algorithm as presented here.
2. The full algorithm without checking for conflicts of smoking status.
3. GP status codes only (i.e. with no codes pertaining to administration or therapy).

The agreement between the WHS data and the three GP data algorithms was estimated using the Kappa statistic [26]. These were done for a two status solution (current smoker, current non-smoker) and a three status solution (current smoker, ex-smoker, never-smoker). For the three status solutions the data in Tables 3, 4 and 5 were used directly. For the two status solutions, data for ex-smoker and never-smoker were amalgamated in each dataset in the above tables.

**Table 3:** Contingency table for the comparison between WHS 2013 ﻿and 2014﻿ data and the same individuals in GP data. Full algorithm as presented here

	WHS				Totals
GP	Missing data	smoker	Ex-smoker	Never smoker	
Missing data	<5	89	120	199	413
Smoker	17	1040	426	209	1692
Ex-smoker	34	130	1449	1104	2717
Never smoker	16	42	186	1770	2014
Totals	72	1301	2181	3282	6836

**Table 4:** Contingency table for the comparison between WHS 2013 and 2014 data and the same individuals in GP data. Full algorithm but without checking for previous smoking status

	WHS				Totals
GP	Missing data	smoker	Ex-smoker	Never smoker	
Missing data	<5	99	120	199	423
Smoker	16	1017	452	236	1721
Ex-smoker	22	112	1108	126	1368
Never smoker	29	73	501	2721	3324
Totals	72	1301	2181	3282	6836

**Table 5:** Contingency table for the comparison between WHS 2013 and 2014 data and the same individuals in GP data. Nearest status code

	WHS				Totals
GP	Missing data	smoker	Ex-smoker	Never smoker	
Missing data	<5	100	124	203	423
Smoker	10	985	194	20	1209
Ex-smoker	25	133	1323	143	1624
Never smoker	32	83	540	2916	3571
Totals	72	1301	2181	3282	6836

### Comparison of prevalence estimates

A comparison was made between annual prevalence estimated from the GP data for men and women using the new algorithm and published values from the Welsh Health Survey over the period 2007 to 2015 [27]. The prevalence calculated from our algorithm from the GP data allocated RELAPSED_SMOKER and LIKELY_SMOKER to SMOKER. Note that these are aggregated annual values from the GP data and the WHS data separately and are not based on linked data. Both represent the population of people aged 16 and above.

## Results

### Prevalence of smoking status

**Fig. 3:**
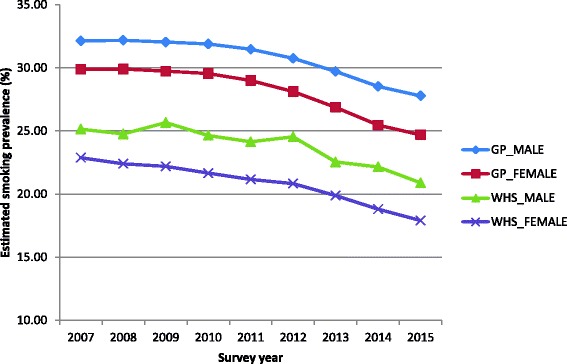
Estimates of smoking prevalence from the Welsh Health Survey and from the GP data, for people aged 16 and over

A comparison between WHS smoking prevalence of people aged 16 and over between 2007 and 2015 with prevalence calculated from our algorithm from the GP data (Fig. 3) indicated GP calculated values to be approximately 7% higher than WHS values. The two datasets both reflect the expected fall in prevalence across the years.

### WHS 2013/2014 and GP smoking status

Comparisons between the status of individuals in GP data and in the WHS 2013 and 2014 data are presented in Tables 3–5.

There are low proportions of missing data; 1.1% missing from WHS data and 6% from GP data (Tables 3–5).

Values of Kappa are presented (Table 6) for a series of comparisons between the WHS and GP datasets. For both the two status and three status solutions, the highest agreement is with the nearest status algorithm, followed by the full algorithm without temporal checking. The lowest agreement is found with the full algorithm. However, all are classified as “good” agreement, according to Altman’s classification [26] (κ between 0.61 and 0.80), except for the three status full algorithm where agreement is “moderate” (κ between 0.41 and 0.60).

**Table 6:** Kappa statistic applied to the contingency tables above for a two status and three status solution. Kappa statistic with lower and upper 95% confidence intervals in parentheses

GP data algorithm	Two status solution	Three status solution
Full algorithm	0.64 (0.62, 0.66)	0.50 (0.48, 0.52)
Full algorithm without temporal checking	0.61 (0.59, 0.64)	0.62 (0.61, 0.64)
Nearest status	0.78 (0.76, 0.80)	0.71 (0.70, 0.73)

One notable difference in classification between the full algorithm and WHS data (Table 3) is the substantial number of people who are classified in the WHS as never-smokers and are classified by the algorithm as ex-smokers. This is a result of past indications of smoking in the GP record. This amounts to 34% of those classified in the WHS as never-smokers. In contrast, the results from the algorithm which do not take previous smoking status into account (Table 4) show only 3.8% of WHS never-smokers were classified as ex-smokers. Again, for the case where the nearest status is used and no previous status is taken into account, (Table 5), 4.3% of WHS never-smokers were classified as ex-smokers.

The full algorithm (Table 3) classifies 8.6% of WHS ex-smokers as never-smokers. The algorithm without consideration of previous status (Table 4) assigns 23% of these WHS ex-smokers as never-smokers because it does not account for previous evidence of smoking. The result with the nearest status algorithm is similar; the proportion is 26%.

There is a smaller effect in the reclassification as smokers of 20% of those classified by the WHS as ex smokers (Table 3). Again this is the result of evidence of smoking activity around the time of the survey.

The number of smokers in the WHS data is 1301. This is 7% greater than those detected by status codes alone (Table 5). However, the full algorithm and the full algorithm without previous status checking detect 30% and 32% more smokers respectively than the WHS data.

## Discussion

The comparison of smoking prevalence at the annual timescale between the WHS and the GP data (Fig. 3) shows some differences between the two datasets. For both men and women the GP estimates are between 6 and 8% higher than the WHS estimates in each year between 2007 and 2015. Our algorithm will assign some people as smokers who may have self-reported as ex-smokers. This may be a result of evidence that they are currently in receipt of nicotine replacement or other therapy or it may be an indication that they had several quit attempts and that the final status is unclear and defaults to smoking. Both surveys clearly lie parallel to each other showing the expected decline in prevalence over this period. We emphasise that we do not consider either GP data or WHS data as a gold standard either at the individual or at the aggregate level and that the truth probably lies somewhere between the two estimates.

We measured the similarity between the various classifications of GP data and the WHS data using the Kappa statistic (Table 6). The closest match is with the two status solution using the closest status algorithm. This algorithm only uses smoking status codes and when used at the closest date to the WHS survey matches more closely (κ = 0.78). The inclusion of smoking cessation therapies is one reason for the increase in numbers of smokers in the full algorithm with temporal adjustment. We consider it to be self-evident that these people should be treated as smokers unless these codes are followed by an ex-smoker code. This, however, reduces the agreement between the WHS survey and the GP derived status. Similarly, the reclassification of some people as ex-smokers rather than never-smokers in the full algorithm as a result of past evidence of smoking will lead to a loss of agreement between the full algorithm and full algorithm without temporal checking. This serves to highlight our contention that neither of these data sources can be considered as an absolute gold standard. There is a possibility that some people may continue to use nicotine replacement in the long-term and are not actively smoking. The usual assumption is that eventually people will relinquish both smoking and nicotine use.

The inclusion of information pertaining to cessation as an indicator of current smoking status is an integral part of the algorithm. We have included prescription records of nicotine replacement therapy (NRT), varenicline and bupropion – all licensed pharmacotherapies for cessation. We have not included use of nortriptyltine or clonidine, both of which have been suggested as effective in smoking cessation [28], since they are not specific for this treatment and not licensed in the UK for this purpose. We must note that many people may use over the counter NRT in order to quit. However, in Wales, contrasting with England, more people may use prescriptions because they are free of charge. Also, there may be people using e-cigarettes as a cessation aid, but this is not well recorded in GP data.

Others have considered use of smoking cessation therapy as indicative of smoking status. Prescriptions for NRT and bupropion as well as cessation administration codes were used to indicate smoking status in a comparison between data from the Clinical Practice Research Datalink (CPRD) data and a national health survey [16]. In a comparison between the General Practice Research Database (GPRD) and a sample of patients with inflammatory bowel disease, the authors did not use NRT prescription as a surrogate because some prescribed it as a therapy for inflammatory bowel disease [29] and the population selected for the questionnaire was drawn from patients with this condition. Note that these two GP data sources here are essentially the same as GPRD is a forerunner of CPRD.

CALIBER [30] is a platform for using linked electronic health records for translational research. A key component of the platform is the pre-processing of data from the various component health record types, to yield research-ready metadata. Smoking status has been processed in this way and includes cessation therapy in the identification of smokers. The complete methodology and code lists for the CALIBER smoking algorithm are available from the authors.

There are two comparison studies investigating GP data on smoking status with self-reported questionnaires at the individual level. In these studies, questionnaires were sent to patients and these were compared with the data already documented by the GP [23, 29]. The first of these studies, constructed on 890 questionnaires [23], showed a moderate agreement between the sources (κ = 0.50) with the principal discrepancy being the conflict between never- and ex-smokers. The second study [29], comparing GP electronic data and patient notes with questionnaires from 1,400 individuals observed a higher level of agreement (κ = 0.83). Both of these studies used data from the mid 1990s. The use of computers in general practice at that time was relatively new and long series of data for patients had not yet accumulated. It seems likely that had more data accumulated, more of the self-reported ex-smokers would have had GP data to corroborate this. In contrast, the WHS had many patients who self-reported as never smokers and had GP data suggesting they had smoked previously. In the first study [25] 36% of those who self-reported as ex-smokers and had GP data were still classified by the GP as never-smokers, even after those with past evidence of smoking had been reclassified as ex-smokers. In the same study, 5.2% of those who self-reported as never-smokers and had GP data were classified by the GP as ex-smokers. In comparison, our WHS study highlighted differences of 10.8 and 35% respectively.

An additional approach is the comparison of GP records with national survey data [16, 29, 31]. Comparisons were made between Clinical Practice Research Datalink (CPRD) data and Health Survey for England surveys between 2007 and 2011 [16]. Differences between the data sources in estimates of current smoking were less than 1% in all years. Former smoking was underestimated in CPRD by 2-7% but this underestimation had reduced between 2007 and 2011. A comparison between GPRD and the 1996 General Household Survey (GHS) [29] illustrated that the GPRD estimate of current smokers was 79% of the GHS estimate. Conversely, the GPRD estimate of former smokers was notably lower at only 29% of the GHS estimate. An additional large scale research collection of GP data, THIN, was compared with GHS surveys between 2000 and 2008 [32]. Over this period, the agreement between THIN and the GHS has improved, so that in 2000, the THIN predicted prevalence of current smokers was around 74% of the GHS estimate, by 2008 the estimates were within 1%. For ex-smokers, only 36% of GHS estimated numbers of ex-smokers were recorded in THIN, by 2008 this had increased to 80%. It is impossible now to tell if some databases were over-reporting sub-groups or others were underreporting subgroups but the discrepancies are reducing implying more accurate recording in recent years.

Higher prevalence of smoking in Wales from GP data than for survey data has been reported previously [32]. This was a survey comparing the General Lifestyle Survey for 2000 to 2008 with the THIN database and estimating regional prevalence in Scotland, Wales and nine English regions. Wales was the only region in which this was observed and the authors ascribed this to low numbers.

The literature indicates that smoking status codes have several associated complications. The first of these is the use of the parent code for the smoking status hierarchy (137.. “Tobacco consumption”). The hierarchy includes codes denoting non-smoking, ex-smoking as well as current smoking, however, it is sometimes utilised in isolation without the subsequent codes. Certain GPs [24, 33, 34] only use the parent code to indicate smoking and other GPs used it with a zero value to indicate non-smoking [24]. In our system, GPs often used the parent code but with a value indicating the number of cigarettes smoked, and therefore it is not possible to use these Read codes to indicate non-smoking (i.e. when a zero was entered).

Another problem is allocating never-smoker status to someone after previous codes denoting smoker or ex-smoker. This effect was taken into account by Booth et al. [16] in calculating prevalence of smoking from GP records and comparing them with estimates from the Health Survey for England. The method developed by these authors [16] was suited to population-level comparison, but was not intended to be optimal at the individual level. This was an annual comparison, so the final status for each individual in a particular year was used to determine the annual status.

Both the CALIBER group [30] and Booth et al. [16] applied similar methods to us, however, our methods yielded a greater number of inferred output categories of potential value. These are completely described and publically available. We believe they are more appropriate for assigning smoking status at the individual level.

### Strengths and limitations

Several studies have compared GP collected data with various surveys. These are usually done from the point of view of validating GP data against a survey. In these studies, the survey is considered as a “gold standard” and the object of the study is to validate the particular GP database in terms of being able to reproduce prevalence values. Thus, for example, THIN data were compared with the General Household Survey [31], with the General Lifestyle Survey [32] and CPRD [16] was compared with the Health Survey of England. In these cases, comparisons were made on an aggregate level not an individual level as in our case.

We have taken the view that the two datasets are different and complementary. The HSW data are collected in a standardized way at a single time, but the GP data are collected in a more ad hoc way over a much longer period. Also, the health survey is designed to be a representative sample of the population, geographically, socioeconomically and with regard to age, whereas the GP sample is not structured in this way.

The primary purpose of our person-level comparison was not to confirm or compare prevalence rates, but to compare the representation of the smoking status of an individual.

In recent years, since 2006 and the introduction of the Quality and Outcomes Framework (QOF) into general practice, GP data have been collected more systematically, but still have a lower degree of completeness than health survey data. However, GP data give a temporal dimension to the question of smoking status. It is possible to reassign some self-reported cases of never smoking to ex smoking and to a lesser extent, some cases of ex-smoking to smoking. For reasons given above in the Methods section, smoking data previously recorded by GPs may be considered definitive and further questioning may not be done. Other people will be requestioned regularly. So there will not always be a very recent coding of status. Finally we must remember that there is always a degree of uncertainty in any dataset about smoking status, that respondents may feel themselves under pressure to understate the extent to which they smoke. It is not possible to be certain to what extent these two datasets differ in this regard and both must be seen as essentially self-reports. The gold standard in reporting smoking status is some form of biochemical test [17]. A systematic review of the use of one such biomarker, cotinine to confirm self-report [35], concludes that the degree of the underestimation of self-report is highly dependent on the medium in which cotinine was measured. The discrepancy between reported and measured prevalence was lower, and the sensitivity was higher when salivary cotinine was used. So there are many difficulties in standardization which the Russell standard addresses using carbon monoxide rather than cotinine [17]. In any case, neither of our data sources has been confirmed in this way.

#### Future work

In the absence of an accepted “gold standard” we believe that an alternative approach to validating this algorithm would be to apply this algorithm, and other alternative published algorithms, to a range of cohort studies. This will enable us to see which algorithm for smoking status is the most predictive in identifying known effects of smoking on health. We will use the algorithms in a series of studies as a covariate and as a confounding variable. However, we believe that this would entail a significant body of work and is outside the scope of this paper, but will be conducted in a future research project.

We also hope to investigate the non-randomness of missingness in GP data. We know that particular groups of people are less likely to visit GPs and have their smoking status recorded. We will be able to circumscribe the parameters of missingness by imputing WHS patient level data and thus be able to account for this in studies in which GP data are used.

The role of e-cigarettes as an aid to smoking cessation is increasing and this is likely to continue in the future. We expect that their use will be more often coded in GP data. The WHS started recording their use in the 2015 survey. This is something we may need to take into account in future work.

## Conclusions

This work aims to improve the accuracy of assigning smoking status utilising GP records. We present the Read codes used to define different categories of smoking status and cessation, as well as investigating the entire history of a patient to identify the current smoking status of the patient at a set time.

The algorithm presented in this paper and the GP clinical systems referred to all partially use V2 Read codes [36] and has direct applicability to several of the UK based research databases CPRD, GPRD [37], THIN [38], Doctors’ Independent Network (DIN) [39], and QRESEARCH [40]).

## Additional file

Additional file 1: Table S1. List of Read codes used in this study. A list of the Read codes used in the study with the starting category for each code. (DOCX 25 kb)

## Abbreviations

CIPHER: Centre for Improvement in Population Health through e-Records Research
CPRD: Clinical Practice Research Datalink
DIN: Doctors’ Independent Network
GHS: General Household Survey
GP: General Practitioner
GPRD: General Practice Research Database
NRT: Nicotine replacement therapy
QOF: Quality Outcomes Framework
SAIL: Secure Anonymised Information Linkage
SQL: Structured query language
THIN: The Health Improvement Network
WDS: Welsh Demographic Service
WHS: Welsh Health Survey
