# Supplementary material for: Development of an algorithm for determining smoking status and behaviour over the life course from UK electronic primary care records
Source: BMC Med Inform Decis Mak. 2017 Jan 5;17:2. doi: 10.1186/s12911-016-0400-6 (PMC5217540; doi:10.1186/s12911-016-0400-6)
Supplement: Additional file 1: Table S1. — List of Read codes used in this study. A list of the Read codes used in the study with the starting category for each code. (DOCX 25 kb) [file 12911_2016_400_MOESM1_ESM.docx]

Additional file 1 Table S1

List of Read codes used in this study.

Some codes (suffixed with %) have been presented as a set of codes under a wildcard.

Categories are S smoker, E ex-smoker, N never-smoker

| **Read codes** | **Description** | **Category** | **Notes** |
| --- | --- | --- | --- |
| 1371. | Never smoked tobacco | N |  |
| 9kn.. | Non-smoker annual review - enhanced services administration | N |  |
| 137K. | Stopped smoking | E |  |
| 137L. | Current non-smoker | E |  |
| 137N. | Ex pipe smoker | E |  |
| 137O. | Ex cigar smoker | E |  |
| 137S. | Ex smoker | E |  |
| 137T. | Date ceased smoking | E |  |
| 1377. | Ex-trivial smoker (< 1 per day) | E |  |
| 1378. | Ex-light smoker (1 - 9 per day) | E |  |
| 1379. | Ex-moderate smoker (10 - 19 per day) | E |  |
| 137A. | Ex-heavy smoker (20 - 39 per day) | E |  |
| 137B. | Ex-very heavy smoker (40 + per day) | E |  |
| 137F. | Ex-smoker - amount unknown | E |  |
| 137i. | Ex tobacco chewer | E |  |
| 137j. | Ex-cigarette smoker | E |  |
| 137K0 | Recently stopped smoking | E |  |
| 9km.. | Ex-smoker annual review - enhanced services administration | E |  |
| 13p4. | Smoking free weeks | E |  |
| 137l. | Ex roll-up cigarette smoker | E |  |
| 745H% | (Various) Smoking cessation therapy | S |  |
| du3% | (Various) Nicotine replacement therapy | S |  |
| du6% | (Various) Bupropion | S |  |
| du7% | (Various) additional nicotine replacement therapy | S |  |
| du8% | (Various) Varenicline | S |  |
| du9% | (Various) Nicotine withdrawal products | S |  |
| E251% | (Various) tobacco dependence | S |  |
| 137.. | Tobacco consumption | S | All with EVENT_VAL greater than 0 |
| 137Z | Tobacco consumption NOS | S |  |
| 137X. | Cigarette consumption | S |  |
| 137Y. | Cigar consumption | S |  |
| 137E. | Tobacco consumption unknown | S |  |
| 137g. | Cigarette pack years | S |  |
| 1372. | Trivial smoker - < 1 per day | S |  |
| 1373. | Light smoker - 1-9 per day | S |  |
| 1374. | Moderate smoker - 10-19 per day | S |  |
| 1375. | Heavy smoker - 20-39 per day | S |  |
| 1376. | Very heavy smoker - 20-39 per day | S |  |
| 137a. | Pipe tobacco consumption | S |  |
| 137b. | Ready to stop smoking | S |  |
| 137C. | Keeps trying to stop smoking | S |  |
| 137c. | Thinking about stopping smoking | S |  |
| 137e. | Smoking restarted | S |  |
| 137G. | Trying to give up smoking | S |  |
| 137H. | Pipe smoker | S |  |
| 137J. | Cigar smoker | S |  |
| 137M. | Rolls own cigarettes | S |  |
| 137P. | Cigarette smoker | S |  |
| 137Q. | Smoking started | S |  |
| 137R. | Current smoker | S |  |
| 137V. | Smoking reduced | S |  |
| 137D. | Admitted tobacco cons untrue ? | S |  |
| 137d. | Not interested in stopping smoking | S |  |
| 137f. | Reason for restarting smoking | S |  |
| 137h. | Minutes from waking to first tobacco consumption | S |  |
| 6791. | Health ed. - smoking | S |  |
| 67910 | Health education - parental smoking | S |  |
| 137m. | Failed attempt to stop smoking | S |  |
| 13p.. | Smoking cessation milestones | S |  |
| 13p0. | Negotiated date for cessation of smoking | S |  |
| 13p8. | Lost to smoking cessation follow-up | S |  |
| 38DH. | Fagerstrom test for nicotine dependence | S |  |
| 67A3. | Pregnancy smoking advice | S |  |
| 67H1. | Lifestyle advice regarding smoking | S |  |
| 67H6. | Brief cessation for smoking cessation | S |  |
| 8B2B. | Nicotine replacement therapy | S |  |
| 8B3f. | Nicotine replacement therapy provided free | S |  |
| 8B3Y. | Over the counter nicotine replacement therapy | S |  |
| 8BP3. | Nicotine replacement therapy provided by community pharmacis | S |  |
| 8CAg. | Smoking cessation advice provided by community pharmacist | S |  |
| 8CAL. | Smoking cessation advice | S |  |
| 8CdB. | Stop smoking service opportunity signposted | S |  |
| 8H7i. | Referral to smoking cessation advisor | S |  |
| 8HBM. | Stop smoking face to face follow-up | S |  |
| 8HkQ. | Referral to NHS stop smoking service | S |  |
| 8HTK. | Referral to stop-smoking clinic | S |  |
| 8I2I. | Nicotine replacement therapy contraindicated | S |  |
| 8I2J. | Bupropion contraindicated | S |  |
| 8I39. | Nicotine replacement therapy refused | S |  |
| 8I3M. | Bupropion refused | S |  |
| 8I6H. | Smoking review not indicated | S |  |
| 8IAj. | Smoking cessation advice declined | S |  |
| 8IEK. | Smoking cessation program declined | S |  |
| 8IEM. | Smoking cessation drug therapy declined | S |  |
| 9hG.. | Exception reporting: smoking quality indicators | S |  |
| 9hG0. | Excepted from smoking quality indicators: Patient unsuitable | S |  |
| 9hG1. | Excepted from smoking quality indicators: Informed dissent | S |  |
| 9kc.. | Smoking cessation - enhanced services administration | S |  |
| 9kc0. | Smoking cessatn monitor template complet - enhanc serv admin | S |  |
| 9ko.. | Current smoker annual review - enhanced service admin | S |  |
| 9N2k. | Seen by smoking cessation advisor | S |  |
| 9N4M. | DNA - did not attend smoking cessation clinic | S |  |
| 9Ndg. | Declined consent for follow-up by smoking cessation team | S |  |
| 9NdV. | Consent given follow-up after smoking cessation intervention | S |  |
| 9NdW. | Consent given for smoking cessation data sharing | S |  |
| 9NdY. | Declin cons follow-up evaluation after smoking cess interven | S |  |
| 9NdZ. | Declined consent for smoking cessation data sharing | S |  |
| 9NS02 | Referral for smoking cessation service offered | S |  |
| 9OO.. | Attends stop smoking monitor admin | S |  |
| 9OO1. | Attends stop smoking monitor | S |  |
| 9OO2. | Refuses stop smoking monitor | S |  |
| 9OO3. | Stop smoking monitor default | S |  |
| 9OO4. | Stop smoking monitor 1st lettr | S |  |
| 9OO5. | Stop smoking monitor 2nd lettr | S |  |
| 9OO6. | Stop smoking monitor 3rd lettr | S |  |
| 9OO7. | Stop smoking monitor verb.inv. | S |  |
| 9OO8. | Stop smoking monitor phone inv | S |  |
| 9OO9. | Stop smoking monitoring delete | S |  |
| 9OOA. | Stop smoking monitor check.done | S |  |
| 9OOB. | Stop smoking invitation short message service text message | S |  |
| 9OOB0 | Stop smoking invitation first SMS text message | S |  |
| 9OOB1 | Stop smoking invitation second SMS text message | S |  |
| 9OOB2 | Stop smoking invitation third SMS text message | S |  |
| 9OOZ. | Stop smoking monitor admin.NOS | S |  |
| E023. | Nicotine withdrawal | S |  |
| J0364 | Tobacco deposit on teeth | S |  |
| SMC.. | Toxic effect of tobacco and nicotine | S |  |
| TJHy2 | Adverse reaction to nicotine | S |  |
| U6099 | [X] Bupropion causing adverse effects in therapeutic use | S |  |
| ZV4K0 | [V] Tobacco use | S |  |
| ZV6D8 | [V] Tobacco abuse counselling | S |  |
| 13p5. | Smoking cessation programme start date | S |  |
| 9ko.. | Current smoker annual review - enhanced service admin | S |  |
